# Experiences of feeding young children with Down syndrome: parents’ and health professionals’ perspectives

**DOI:** 10.1080/20473869.2023.2269321

**Published:** 2023-12-06

**Authors:** Silvana E. Mengoni, Bobbie Smith, Helena Wythe, Samantha L. Rogers

**Affiliations:** 1 Centre for Health Services and Clinical Research, University of Hertfordshire, Hatfield, UK; 2 Department of Psychology, Sport and Geography, University of Hertfordshire, Hatfield, UK; 3 Centre for Research in Public Health and Community Care, University of Hertfordshire, Hatfield, UK

**Keywords:** Down syndrome, feeding, eating, mealtimes, breastfeeding

## Abstract

**Background:**

Children with Down syndrome are commonly reported to experience feeding problems in the early years. This study aimed to explore and synthesise the experiences of feeding young children with Down syndrome from parents and professionals, and the support needed and received during this time.

**Methods:**

Eight mothers and twelve healthcare professionals took part in semi-structured interviews. All participants had, or had supported, a child(ren) with Down syndrome aged 0–5 years.

**Results:**

Reflexive thematic analysis resulted in two themes and seven subthemes. Mothers had clear feeding goals and adapted their journeys to meet their child’s individual needs, with support from professionals and peers. Professionals could empower parents by building confidence and offering proactive support, although a lack of knowledge about Down syndrome and difficulties accessing support undermined mothers’ confidence in services.

**Conclusions:**

Breastfeeding and family mealtimes held significant value to mothers, and specialist and trusted support may be needed to help families achieve these goals.

## Introduction

Down syndrome is the most common genetic cause of intellectual disability, typically caused by an extra copy of chromosome 21, with a live birth prevalence rate of approximately 1–1.2 per 10,000 (Wu and Morris 2013, de Graaf *et al.* 2015). Infants and young children with Down syndrome may experience feeding problems, including challenges with breastfeeding (Magenis *et al.* 2022, Zhen *et al.* 2021) and eating solid food (Anil *et al.* 2019, Cochran *et al.* 2022, Rogers *et al.* 2022). It is crucial to understand early feeding to reduce the potential long-term impact of long-term issues such as difficulty chewing, swallowing, food neophobia and obesity (Chenbhanich *et al.* 2019, Ravel *et al.* 2020, Cañizares-Prado *et al.* 2022).

Exclusive breastfeeding is recommended for the first six months of life, and continued breastfeeding is recommended until a child is at least two years, due to the beneficial long-term effects such as protecting against obesity and infections (World Health Organization 2022). As children with Down syndrome are more likely to experience such health issues, breastfeeding may be particularly important (Barros da Silva *et al.* 2019). Rates of breastfeeding for children with Down syndrome vary across studies and countries (Magenis *et al.* 2022). There is limited data in the United Kingdom (UK), although Williams *et al.* (2022) found similar rates of exclusive breastfeeding for children with Down syndrome compared to the general population, with 21% being exclusively breastfed at 6 wk compared to 23% respectively. However, mothers of infants with Down syndrome often report challenges with breastfeeding, including illness or hospitalisation, difficulty with sucking and swallowing, weight loss, perceived lack of milk supply and post-natal mental health. These difficulties are often related to issues related to Down syndrome, for example cardiac problems, and a perceived lack of support from health professionals (Barros da Silva *et al.* 2019, Cartwright and Boath 2018, Magenis *et al.* 2022, Zhen *et al.* 2021).

Introduction of complementary foods to children with Down syndrome may occur later than for typically developing children (Mohamed *et al.* 2013, Rogers *et al.* 2022), with variable guidance and support provided to parents (Cochran *et al.* 2022). Children with Down syndrome may experience problems with textured food, swallowing and self-feeding, and these problems may be greater than typically developing peers (Collins *et al.* 2003, Mohamed *et al.* 2013, Anil *et al.* 2019).

Families of children with Down syndrome value shared decision-making, reliable and timely information and emotional support (Skelton *et al.* 2021), but often report an unmet need for support from health professionals, who can be seen to lack knowledge about Down syndrome (Minnes and Steiner 2009, Marshall *et al.* 2015, Douglas *et al.* 2017). Cochran *et al.* (2022) found that there was limited training and Down syndrome-specific guidance available for professionals supporting families with the introduction of complementary food. However, this American-based study did not extend beyond this to explore professionals’ views on supporting breastfeeding, which has been reported as a significant area of unmet need by parents.

To gain a holistic understanding of early feeding experiences and support, it is important to explore if and how the perspectives of parents and professionals align and what the implications are for practice, which is missing from the existing literature. Therefore, this study aimed to explore the experiences of feeding children with Down syndrome in the early years, feeding problems encountered and provision of support from parents and professionals, and to identify areas of, and reasons for, commonalities and tension.

## Materials and methods

### Design

**Figure 1.**
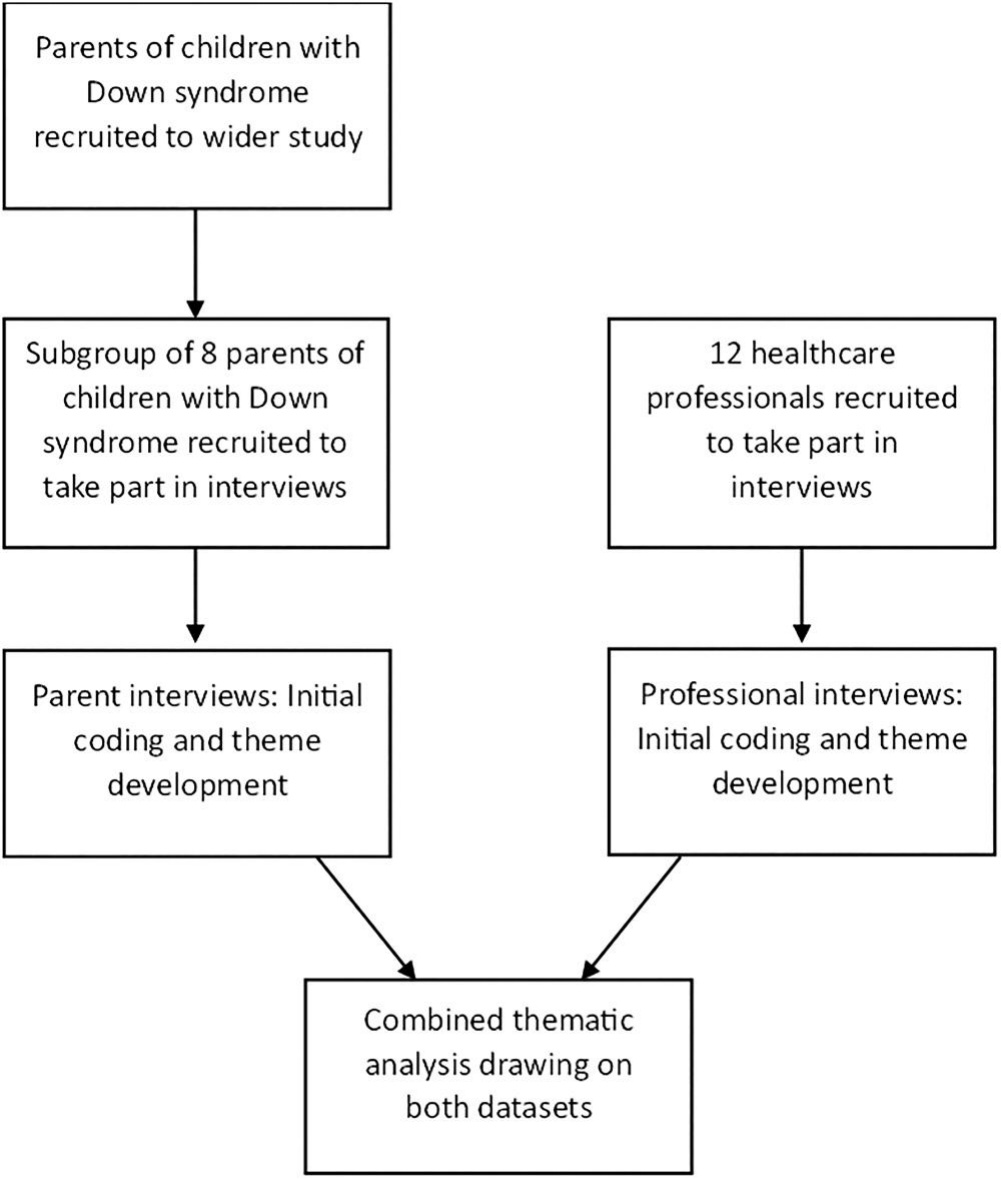
An outline of the study’s methodology.

Semi-structured individual interviews were conducted and reflexive thematic analysis was used (Braun and Clarke 2006, 2022), with an inductive experiential approach to centre and draw meaning across participants’ experiences. Figure 1 outlines the methodology of the study.

### Participants

Parents were recruited from a wider study (Rogers *et al.* 2022) and eligibility criteria included being over the age of 18 years, living in the UK and having a child with Down syndrome aged 6 months to 5 years. Eight mothers took part in the present study, and their children with Down syndrome were aged 9–52 months with a mean age of 28 months (five female, three male). All mothers had at least one older child without Down syndrome. Demographic details for the mothers and children are shown in Table 1.

**Table 1.** Demographics of mothers and children.

	Mean (SD) / *N* (%)
Child’s age (months)	28.25 (15.00)
Child’s number of siblings	1.25 (0.71)
Mother’s age (years)	42 (3.5)
Mother’s BMI	22.88 (3.08)
Mother and child dyad ethnicity	
White British	6 (75%)
White Other	1 (12.5%)
Mixed	1 (12.5%)
Mother’s highest level of education	
Further secondary education (16–18 years)	1 (12.5%)
Degree	4 (50%)
Further degree	3 (37.5%)

Healthcare professionals in two NHS Trusts in Hertfordshire in England with experience of supporting families of children with Down syndrome aged 0–5 years with feeding issues were invited to take part. Twelve healthcare professionals participated and all were female. There were eight health visitors, two speech and language therapists, one occupational therapist and one midwife. In the UK, health visitors are qualified nurses or midwives with additional training, and they support families during the early years of a child’s life. Details for each participant across both groups are shown in Table 2, along with details about their child/children they supported.

**Table 2.** Overview of participants.

Participant code	Role (professionals only)	Details of their child with Down syndrome / children with Down syndrome supported in their current role
Mother1		Daughter with Down syndrome aged 9 months; currently breastfeeding and introduced solids at 6 months
Mother2		Daughter with Down syndrome aged 13 months; currently breastfeeding and introduced solids at 6 months
Mother3		Boy with Down syndrome aged 47 months; breastfed for 6 months and introduced solids at 4.5 months
Mother4		Boy with Down syndrome aged 15 months; currently breastfeeding and introduced solids at 6.5 months
Mother5		Girl with Down syndrome aged 31 months; breastfed for 8 months and introduced solids at 8 months
Mother6		Boy with Down syndrome aged 52 months; expressed breastmilk for 10 wk and introduced solids at 6 months
Mother7		Girl with Down syndrome aged 37 months; breastfed for 18 months and introduced solids at 7 months
Mother8		Girl with Down syndrome aged 25 months; breastfed for 24 months and introduced solids at 6 months 3 wk.
Professional1	Health visitor	Supported two children with Down syndrome
Professional2	Health visitor	Supported two children with Down syndrome
Professional3	Health visitor	Supported three children with Down syndrome
Professional4	Health visitor	Supported two children with Down syndrome
Professional5	Health visitor	Supported ten children with Down syndrome
Professional6	Health visitor	Supported one child with Down syndrome
Professional7	Health visitor	Supported three children with Down syndrome
Professional8	Health visitor	Supported four children with Down syndrome
Professional9	Speech and language therapist	Supports approximately eight children with Down syndrome per year
Professional10	Speech and language therapist	Supported fifty children with Down syndrome
Professional11	Midwife	Supports one-two children with Down syndrome per year
Professional12	Occupational therapist	Supported one child with Down syndrome

### Interview schedules

The semi-structured interview schedules included open-ended questions and prompts. Both interview schedules were developed drawing on existing literature and feedback from a parent of a young person with Down syndrome. Mothers were asked about their plans for, and experiences of, milk feeding and introducing complementary foods, along with mealtimes more generally. Mothers were also asked about their experiences of support and any support needs.

The majority of the interviews with mothers took place first and informed the interview schedule for the professionals, which included experiences and challenges of providing feeding support, training received and their support needs.

### Procedure

Ethical approval was obtained from University of Hertfordshire and governance approval was obtained from the Health Research Authority and the participating NHS Trusts.

After participation in a wider study (advertised *via* social media, support groups and national charities), parents were invited to participate in an interview. Interested participants were provided with further information and informed consent was given for this study. Interviews took place from February 2018 to February 2019 in the participants’ home or by telephone depending on participant preference and geographical location. Interviews lasted 15–20 min and were audio-recorded. Interviews were likely shorter than the professionals’ interviews as other data collection (not reported here) also took place during this visit.

Study information was shared in the two NHS Trusts aimed at services that provided support to children with Down syndrome aged 0–5 years for feeding and eating. Information was cascaded by managers and interested healthcare professionals contacted the researchers, who arranged an appointment to discuss further, take informed consent and conduct the interview. Interviews took place from December 2018 to May 2019 and were carried out at the NHS Trust, at the University of Hertfordshire or *via* Zoom, depending on the participant’s preference. Interviews lasted 20–70 min and were audio-recorded.

### Analysis

Braun and Clarke’s (Braun and Clarke 2006, 2022) six-phase process for reflexive thematic analysis was followed: familiarization with the dataset; coding; generating initial themes; developing and reviewing themes; refining, defining and naming themes; writing up. All interviews were transcribed intelligent verbatim and were read by the analysts. The analysis took place on NVivo. Initial coding was semantic (e.g. descriptions of feeding problems) and latent (e.g. reflections on why support was perceived as effective).

Analysis was initially undertaken separately for the parent (BS) and professional data (HW), as funding and data collection occurred at different times. SM and SR met with both analysts throughout the initial coding and theme development stage. It was evident that the thematic analysis had significant similarities across the separate parent and professional datasets and that there was analytic value in combining these. SM then reviewed the transcripts, coding and initial themes across both datasets and developed themes and subthemes that captured and synthesized meaning from both parents and professionals. As part of the writing process, themes were further refined, defined and named. Reflective notes and discussions amongst the team were important to interrogate the analysis and facilitate credibility. The analysis is viewed as a result of participants’ stories and the analysts’ interpretation of these experiences and the context they sit within (Braun and Clarke 2022).

## Results

The analysis resulted in two themes and seven subthemes as shown in Table 3.

**Table 3.** An overview of the thematic analysis.

Theme 1: Persevering to meet feeding goals	Theme 2: Empowered families at the centre of a trusted support network
Value of feeding and eating to family life	Building mothers’ confidence
Impact of multiple needs on feeding problems	The parental need for proactive and ongoing support
Meeting feeding goals by adapting plans	A multidisciplinary team is needed to address the myriad feeding issues
	The importance of knowledge and experience of Down syndrome

### Persevering to meet feeding goals

Mothers often had clear feeding goals to breastfeed, use baby-led weaning approaches, and share family mealtimes. Their children’s needs often led to changes in feeding plans and required support from professionals. Therefore, journeys often differed to mothers’ initial plans, but perseverance to navigate these challenges and reach the ultimate destination was key.

#### Value of feeding and eating to family life

Both mothers and professionals spoke about aspects of feeding and eating that were important to families, often breastfeeding and sharing meals. They highlighted the emotional value of breastfeeding for the mother-infant relationship.

‘I mean I am really glad I managed to because it has definitely helped her in terms of her health and our bonding’ Mother7

‘I do think that [breastfeeding] affects how you feel about your baby, so I think it was important, and it made her feel like she was just the same as the other three really’ Professional2 talking about a mother who had breastfed her older children

Mothers expressed happiness around watching their child reach milestones despite their own and others’ expectations, including ‘proving me wrong’ (Mother1). Moving past the introduction of complementary foods phase, many mothers reported that their infants were overall ‘really good eater[s]’ (Mother5). The benefits and enjoyment of engaging in social rituals and valuable family time at mealtimes were highlighted.

‘He loves all the kind of ceremony associated with eating. You know like doing cheers […] and all of that kind of thing… He lays the table for me’ Mother6.

However, it was recognised that mealtimes could cause stress and there was an ongoing need for strategies to promote a calm and positive environment:

‘We try and kind of give an air of everything’s fine and stay calm and relaxed, but obviously it is difficult to be chilled about like she wasn’t taking in as much food as she should have done’ Mother7

‘That is helpful as well, to think about the overall sensory experience of eating and what strategies, you know, if mealtimes become a bit of a battle or a bit of an area of tension, how you can [use] sensory strategies to help kind of calm’ Professional12

#### Impact of multiple needs on feeding problems

The interacting health needs of children often affected milk feeding and introducing complementary foods, although as highlighted by Professional6, ‘it’s case-by-case you know, not all children obviously with Down Syndrome are going to be the same’. However, there were some commonly reported issues, including hospitalisation, illness, fatigue, cardiac issues, reflux and surgery, which often led to breastfeeding challenges. Mother6, who was the only participant not to breastfeed directly and expressed for 12 wk, highlighted the impact of low tone on their journey. During hospitalisation, mothers reported that providing breastmilk was sometimes not a priority of healthcare professionals, which was at odds with their wishes.

'The only thing that I am still quite unhappy about is that when we were in hospital, when he was first born, they like, because I only had colostrum because he had low blood sugar they wanted to give him a formula top up. I really didn’t want him to have it. I wanted him to have donor milk. They wouldn’t give him any donor milk, so I didn’t feel very supported at that point but yeah that was only a couple of days before my milk came in then it was fine.' Mother4

Delayed oral, gross and fine motor skills were described to affect breastfeeding, chewing and self-feeding.

'I give her a spoon and I have a spoon and she tries, she hasn’t got the scoop, she can’t scoop….So she sticks it in and then tries to eat, but I put my hand over her hand and we scoop together. She’s two and she doesn’t really want Mummy feeding her now.' Mother8

Tube feeding was also felt to disrupt feeding. Professionals spoke about prioritising safety when introducing complementary foods to children who had been tube fed, for example giving purees and melt-in-the-mouth foods to reduce the risk of choking. Safety was a particular concern for mothers too, even for children who had not been tube fed, as they were aware that children with Down syndrome typically had more issues with choking.

'Well the tube was left in for quite a long time, too long probably and then they had specialist feeding intervention to get on to solid food and then to be able to eat family meals.' Professional8

'I do worry more about choking with her, although she has not exhibited any difficulties that are any different to what my son had.' Mother1

#### Meeting feeding goals by adapting plans

There was a strong desire from all the mothers in this study to breastfeed and persevering with this plan was important.

'Oh we were planning on breastfeeding… it was the only plan that I was able to stick with.' Mother2

However, a positive breastfeeding experience was considered ‘lucky’ (Mother8). Some mothers also spoke about benefiting from their experience breastfeeding their older children. Mothers and professionals acknowledged the significant benefits of breastfeeding but often spoke about a challenging journey, and the importance of support to navigate this.

'Sometimes they find the bottle a little bit easier because it just pours in but for jaw development and mouth development and the stimulation and the skin-to-skin and the closeness with mum, breastfeeding is the best way to feed the baby.' Professional11

There was often a tension between bottle feeding and mothers’ breastfeeding goals as acknowledged by both groups of participants, and formula feeding was something breastfeeding mothers often worked hard to avoid where possible.

'I think she was advised by the paediatricians that she probably ought to start thinking of formula, but she just thought that’s not going to help her tummy, and her systems are all under a bit more stress, no, I want to keep breastfeeding. And it wasn’t… I felt comfortable with it, it wasn’t a big drop, it was just very slow gains, really.' Professional2 speaking about supporting a mother who wanted to continue breastfeeding

Mothers often spoke about adapting their plans to use finger foods for introducing complementary foods to accommodate delayed motor skills and aspiration risk. Some mothers spoke about experiences with their older children, but they then had very different experiences with their child with Down syndrome.

'It was challenging because we weren’t sure… having had an older child who obviously had gone through the typical route of finger foods and everything. We all of a sudden were kind of faced with these different things, we didn’t know how to kind of go about it.' Mother5

Professionals advised parents about finger foods they could use safely, in line with mothers’ desires to use baby-led methods where possible.

'We have a snack time at the Centre and so that’s really nice then to be able to give that specific advice to that child about encouraging finger foods and which type of finger foods are safe for that child.' Professional9

### Empowered families at the centre of a trusted support network

Emotional, practical and informed support was key to empowering parents. Mothers valued support from professionals, with an emphasis on proactive and ongoing input and Down syndrome-specific expertise, but this was sometimes not within the service provision model, leading to unmet needs for support. Peer support and online information were sometimes sought to fill this gap, but an outstanding need for professional input often remained.

#### Building mothers’ confidence

Emotional support and reassurance enabled mothers to have the confidence to follow their plans, particularly around breastfeeding:

'Mum did need a bit of emotional support to realise that she was making… reassuring though that she was making the right decisions by continuing the breastfeeding, and not being… I think had she been a first time mum maybe she’d had felt she needed to introduce formula, but she didn’t, and I think but she did seek a bit of reassurance on it.' Professional2

'If it hadn’t been for her I would not have breastfed full stop. It would not have happened. Because she put hours in when she came in, she put hours and hours to help her and to teach her and to give me the confidence to keep trying.' Mother7 regarding hospital breastfeeding specialist

Mothers valued being able to check concerns with professionals, to enable themselves to manage their child’s eating and drinking independently. Likewise, professionals valued the opportunity to provide parents with advice and practical support, which would empower them to feed their children appropriately and safely, including breastfeeding positioning and ways of introducing complementary foods.

'I just wanted to make sure that all the alternatives that we had him on like oat milk and rice cakes and gluten free bread and all that she was happy with and she was fine with that, so that was good to get that sign off […]. I think that has given me the confidence really just to know yeah he’s fine, he is getting a good diet, a healthy diet and he is able to choose.' Mother3

'To start with it was like a bit of milk and then a bit of food, then a bit of milk, just sort of trying to encourage him to taste different things, so mum tasting, getting, trying, getting him to try different things from her plate.' Professional5

#### The parental need for proactive and ongoing support

Mothers who felt supported in their feeding journey reflected on this as being lucky, and a feature of Mother2’s positive experience was that professionals were proactive in offering support and ‘they are always contacting me or asking me something or they are coming here to see how she is doing’. However, some mothers spoke about how they often needed to seek out support from professionals, waiting until a problem had occurred, for example:

'I went to baby clinic a few weeks before 6 months and I just sort of said to the health visitor ‘I am supposed to start weaning soon do I?’ and she was like ‘yes you should’ and then the next baby clinic after that I had to sort of say to them ‘look I am trying to do weaning but she is not ready you know I can’t get her to do anything’ and it is only because I said that, that I got any help at all which was a bit of a shame. It would have been nice to have bit more of a conversation perhaps a bit ahead of my starting.' Mother1

Generally, professionals described a tiered approach to support which reflected the experiences of the mothers in our sample, with low-level universal support at first and more intensive support when parents expressed a need for this.

'We always signpost to places like First Steps to Nutrition, Department of Health, any NICE guidelines, you know, the usual sort of routes really. And then if the parent wants, if the parents are asking for extra help or are having difficulties that’s when we would go into the home and you know, try and go at feed times or mealtimes, to try and really work out what the practical problems are.' Professional8

Professionals spoke about ‘episodes of care’ to support specific issues with feeding when needed, whereas parents sometimes had an expectation of more ongoing support. This was perceived to lead to anxiety and unwillingness about discharge.

'His skills were kind of plateauing and the service, again, was moving to this episode of care, so he needed to be discharged and parents were so concerned about the discharge, even though they didn’t want anything specific to work on'. Professional12

Therefore, there was a tension between parents’ need for proactive support and the tiered and episodic approach to support adopted by services.

#### A multidisciplinary team is needed to address the myriad feeding issues

Professionals spoke about providing support in a multidisciplinary team and sharing information with colleagues. From a mother’s perspective, Mother3 highlighted the benefits of having consistent feeding support across different professionals:

'Everyone can do it consistently which is great. In every setting he is having the same kind of support.' Mother3

Professionals recognised the boundaries of their expertise, for example health visitors felt that providing support for feeding was a ‘big part’ of their role (Professional2), and they spoke about referral and signposting to more specialist support when needed based on their professional judgement and experience.

'When things aren’t our specialist field, we just find out who the right people are to ask and go from there.' Professional4

However, mothers spoke about instances where they did not receive help due to their child’s needs not falling within the remit of services, with Mother8 describing speech and language therapists as being ‘very concerned about choking and that was it’. Professionals also spoke about specialist roles and services which did not have sufficient capacity and had significant waiting lists, impacting on timely support and intervention. Professionals acknowledged that sometimes specialist support was not available due to lack of expertise.

‘Those behavioural feeders, we at this point in time don’t have a suitable service to meet the needs of those children because often for those children you need clinical psychology input for the whole behavioural aspect, so we can’t provide that, and they need a high level of support’ Professional10

#### The importance of knowledge and experience of Down syndrome

Parents viewed peer support groups as an instrumental part of their support networks, as did professionals who often signposted parents to these. Support groups fostered a sense of community and belonging, and parents could receive trusted advice from people who shared similar experiences. Mothers also described them as addressing unmet support needs from professionals, particularly around lack of timely support on specific issues.

'They came so infrequently that the next phase had moved on by the time they came to give advice anyway so it wasn’t responsive enough, which is why Facebook and things like that are far more useful.' Mother8 referring to a lack of support from professionals

Parents noted that information from support groups and online forums could sometimes be contradictory and negative. Therefore the support of professionals was still needed. However, a perceived lack of understanding about Down syndrome could lead to reduced trust.

'because of the low tone, you need to teach them to an extent to feel their body and you don’t get that explanation from sort of like your midwives from sort of like all these breastfeeding experts because they….I don’t think they kind of understand that side of Down syndrome.' Mother6

There was a conflict between parents’ desire for professionals with knowledge and experience of Down syndrome, and professionals feeling able to adapt their existing skillset to children with Down syndrome. Professionals typically described children with Down syndrome as having a general delay with feeding in line with their overall development, and Professional10 expressed that therefore the majority of healthcare professionals had ‘enough of an understanding to support with those general developmental difficulties’. Professionals spoke about how they drew on their existing, not necessarily Down syndrome-specific, knowledge and experience, and adapted this, as Professional6 describes in relation to breastfeeding:

'so I was doing what we would normally do to be honest, doesn’t matter that she’s got Down Syndrome, just normal breastfeeding support, checking positioning and attachment, helping mum with different positions that might make it more comfortable, because of obviously she’s got a larger tongue and the shape of her mouth, her jaw, is slightly different, I do think it has made the positioning and attachment slightly more difficult than it would for any other child, so it’s just been a lot of tweaking and things like that.' Professional6

In line with this, there were mixed views from professionals about whether Down syndrome-specific training would be helpful. Some professionals noted the infrequency of having a child with Down syndrome on their caseload, which meant training might not be relevant in the short or even medium-term. Others spoke about how additional training may be useful, including updates to knowledge and how to adapt support.

‘I think maybe some, just a little bit of additional training within the breastfeeding remit, about common problems that particularly Downs babies might have. They won’t be too different maybe from other babies that have difficulties, but I think that’s an area that we have, it is covered but it’s not covered specifically’ Professional8

## Discussion

Common issues affecting feeding and eating in our study included hospitalization, health conditions, swallowing, chewing and delayed motor skills. Despite this, mothers often had clear visions of how they wanted to feed their children, and commonly this was to breastfeed and share family meals. Families took different journeys than those initially envisaged, persevering and adapting to children’s needs, but the underlying goals remained. Timely and accessible support from knowledgeable sources was important to empower families to meet these goals.

Breastfeeding was an important aspect of feeding to the mothers in our study and a common area of support reported by professionals. Issues around hospitalization, cardiac problems, fatigue and low weight gain meant this could be challenging, although the emotional bond of breastfeeding was highly valued. Some mothers reported positive experiences of support, but in line with other studies, others highlighted a lack of breastfeeding knowledge and support (Barros da Silva *et al.* 2019, Cartwright and Boath 2018, Zhen *et al.* 2021). Although dissatisfaction with breastfeeding support is not unique to mothers of children with Down syndrome (Schmied *et al.* 2011) the additional challenges that these families may face mean that effective support is even more crucial.

The introduction of complementary foods was influenced by the child’s motor skills and increased risk of choking, and professionals spoke about the importance of observations and personalized advice to ensure safety. There were some areas of unmet needs, including when and how to introduce complementary foods, which is in line with the variability in support for parents during this period found by Cochran *et al.* (2022).

All mothers in the present study had older children without Down syndrome, and some spoke about the benefit of prior experiences breastfeeding and introducing complementary foods, although noted differences with their child with Down syndrome often due to medical needs or developmental delays. Further research could explore the impact of previous parenting experiences in more detail, and to what extent this influences feeding decisions and success in meeting goals.

Peers can provide information, reassurance and recommendations for services to other families of children with Down syndrome (Douglas *et al.* 2016, 2017) and our study reflected this for feeding support. As some of our mothers highlighted, not all information from online forums was helpful or accurate, and there can be minimal regulation of online feeding support groups (Garcia *et al.* 2019, Regan and Brown 2019). Whilst support groups have an important role to play in supporting families of children with Down syndrome with feeding issues, it is important that families access this alongside professional input.

Mothers valued easy and timely access to professional support and proactive communication. As the professionals in our study described an episode of care approach, i.e. provision of support for specific issues for a time-limited period, there was an evident tension. A key point of contact for parents to navigate services, along with clear and quick referrals for specialist support could help to address this tension (Ryan and Quinlan 2018, Skelton *et al.* 2021).

Mothers placed great value on Down syndrome-specific knowledge and experience and they were critical when this was lacking in the professionals they worked with, in line with previous research (Skelton *et al.* 2021). Professionals placed less emphasis on this as they felt able to adapt their expertise to specific children and specific feeding needs. It may be that a lack of Down syndrome knowledge and experience undermined parents’ confidence in professionals, and/or it may also be that this lack of expertise affected the support that professionals provided and whether this met families’ needs. In a survey about services for children with neurodisability and eating, drinking and swallowing difficulties, health professionals were more likely than parents to report that interventions were effective (Taylor *et al.* 2021). This discrepancy between parents’ and professionals’ expectations and experiences of feeding support requires further exploration.

In line with previous research highlighting a need to increase health professionals’ knowledge and understanding of intellectual disability (Hemm *et al.* 2015), some professionals suggested that guidance about how to adapt support for children with Down syndrome would be helpful. Down syndrome specific guidance about common breastfeeding challenges and solutions and how to adapt introduction to complementary foods may be useful for professionals when working with families (Blixt *et al.* 2019, Cochran *et al.* 2022).

Through the inclusion of parents and professionals, we aimed to triangulate and synthesise experiences of feeding problems and support in young children with Down syndrome. However, the two groups of participants were not discussing the same children, and therefore the feeding problems and contexts are different. Different methodologies could add to the evidence base, for example multi-perspective case studies of individual children or by collecting data from both parents and professionals on a specific feeding issue.

Our professional participants were primarily health visitors and we lacked representation from key professional groups such as paediatricians and lactation consultants, and only one midwife participated. Furthermore, we had limited geographical representation, which is important due to variability in service provision and may limit the transferability of our conclusions. All our parent participants were mothers, despite the research study being open to both mothers and fathers. This is common to parenting and feeding research but has the potential to perpetuate the feelings of exclusion that fathers may already experience regarding parenting their child with Down syndrome (Docherty and Dimond 2018). All mothers breastfed, with one doing so *via* expressed milk, which is a high rate and our findings may not be representative of all milk feeding experiences. Cultural influences affect experiences of milk feeding and complementary feeding, and therefore these results may not be transferable to other cultures (Pak-Gorstein *et al.* 2009). The interviews were carried out before the COVID-19 pandemic during which mothers of children with Down syndrome reported a lack of feeding support, due to reduced face-to-face support and contact with friends and family (Hielscher *et al.* 2022). Future research should focus on whether these issues have been addressed for families in the post-pandemic context.

## Conclusions

Across the general population, families report challenges and support needs with breastfeeding and the introduction of complementary foods (Odom *et al.* 2013, Blixt *et al.* 2019, Garcia *et al.* 2019), and low rates of breastfeeding are a public health concern (Rollins *et al.* 2016). Infants and young children with Down syndrome are more likely to experience health concerns and developmental delays than typically developing children, which impact feeding and eating and therefore require additional support. However, there is an unmet need for support highlighted by this study. There is a tension between services that focus on an episode of care approach, involvement of multiple agencies with different referral routes and universal care pathways with families who value the reassurance of being able to access support as and when they need it from professionals who have expertise in Down syndrome. A care coordination approach may help to address this tension, with families having ongoing access to a key point of contact, with Down syndrome experience, to increase confidence in service provision and ensure appropriate care is accessed when it is needed.

## Data Availability

Data is not available to share. To encourage open responses to a potentially emotional topic, participants were assured that their transcripts would remain confidential, and only anonymised quotes would be shared.
